# The hop-derived compounds xanthohumol, isoxanthohumol and 8-prenylnaringenin are tight-binding inhibitors of human aldo-keto reductases 1B1 and 1B10

**DOI:** 10.1080/14756366.2018.1437728

**Published:** 2018-03-13

**Authors:** Jan Moritz Seliger, Livia Misuri, Edmund Maser, Jan Hintzpeter

**Affiliations:** a Institute of Toxicology and Pharmacology for Natural Scientists, University Medical School Schleswig-Holstein, Kiel, Germany;; b Department of Biology, Tuscany Region PhD School in Biochemistry and Molecular Biology, University of Pisa, Pisa, Italy

**Keywords:** Xanthohumol, 8-prenylnaringenin, aldo-keto reductases, diabetes, tight-binding inhibition

## Abstract

Xanthohumol (XN), a prenylated chalcone unique to hops (*Humulus lupulus*) and two derived prenylflavanones, isoxanthohumol (IX) and 8-prenylnaringenin (8-PN) gained increasing attention as potential anti-diabetic and cancer preventive compounds. Two enzymes of the aldo-keto reductase (AKR) superfamily are notable pharmacological targets in cancer therapy (AKR1B10) and in the treatment of diabetic complications (AKR1B1). Our results show that XN, IX and 8-PN are potent uncompetitive, tight-binding inhibitors of human aldose reductase AKR1B1 (K_i_ = 15.08 μM, 0.34 μM, 0.71 μM) and of human AKR1B10 (K_i_ = 20.11 μM, 2.25 μM, 1.95 μM). The activity of the related enzyme AKR1A1 was left unaffected by all three compounds. This is the first time these three substances have been tested on AKRs. The results of this study may provide a basis for further quantitative structure–activity relationship models and promising scaffolds for future anti-diabetic or carcinopreventive drugs.

## 1. Introduction

**Figure 1.**
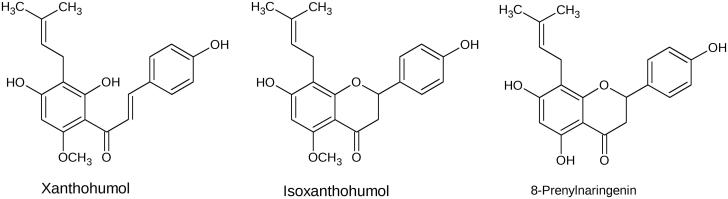
Chemical structures of xanthohumol (XN), isoxanthohumol (IX) and 8-prenylnaringenin (8-PN).

The female inflorescences of hops (*Humulus lupulus*) have long been used in traditional Chinese and Ayurvedic medicine mainly to treat sleep disturbances^1^ and are also known for their antibiotic properties^2^. Among the secondary plant compounds that occur in the resinous inflorescences of *H. lupulus* are prenylated chalcones and other flavonoids^3^. These compounds are well known for their bittering and preserving qualities in the brewing process of beer^3^^,^^4^, as well as for their bioactivity (antibiotic, anti-viral and antioxidant properties)^5–7^. Recently, the prenylated chalcone xanthohumol (XN) has come into focus of biomedical research, due to its versatile bioactive characteristics^8^. Since XN is a unique prenylflavonoid occurring in hops, beer is the only noteworthy dietary source for XN in central Europe. In addition to XN, hops inflorescences also contain flavanones like isoxanthohumol (IX) and 8-prenylnaringenin (8-PN)^9^ (Figure 1), but at 10- to 100-fold lower concentrations than XN. The main metabolic routes for XN, IX and 8-PN have been characterised extensively, both, *in vitro* by using human and rat liver microsomes ^3^^,^^10–12^ and *in vivo*, in rat models^13^^,^^14^. XN is subject to spontaneous conversion into IX via intramolecular Michael addition^15^. IX, in contrast, undergoes an enzymatic *O*-demethylation via hepatic CYP1A2 to 8-PN, which has been described as the most potent phytoestrogen found in nature^5^^,^^16^. All three substances are currently under investigation due to their anti-diabetic^17^^,^^18^, anti-carcinogenic^3^^,^^8^^,^^19^ and antioxidant properties^20^. Moreover, XN has been shown to have anti-HIV traits^21^^,^^22^.

The pathogenesis of several inflammatory conditions, such as diabetes, cancer and sepsis, have in many cases been linked to enzymes of the aldo-keto reductase (AKR) superfamily^23–29^. Among them, AKR1A1, also known as human aldehyde reductase, is involved in the reduction of biogenic and xenobiotic carbonyl group containing compounds, such as the cytotoxic lipid peroxidation-derived aldehydes 4-ONE and 4-HNE^30^^,^^31^ and the chemotherapeutic drugs doxorubicin (DOX) or daunorubicin (DAUN)^32^. It is widely expressed in most tissues, with particularly high levels reported in the cerebrum, liver and kidneys^33^.

The same applies for the homologous enzyme AKR1B1 (60% sequence identity to AKR1A1), commonly known as human aldose reductase^34^. AKR1B1 mediates the first step of the “polyol pathway” by reducing glucose to sorbitol through a NADPH-dependent reaction. Further, sorbitol is oxidised to fructose by sorbitol dehydrogenase, using NAD^+^ as a co-factor^35^^,^^36^. Under normoglycemic conditions only 3% of total glucose is converted into sorbitol through the polyol pathway. Under hyperglycemic conditions, however, the flux of glucose through this metabolic pathway may increase up to 10-fold (30%), which might finally lead to accumulation of excess sorbitol^35^^,^^37–39^. The resulting osmotic stress and imbalances of the pyridine nucleotide redox status decrease the cell’s antioxidative capabilities. In diabetes mellitus, this promotes the formation of advanced glycation end products (AGEs)^34^^,^^40^, which themselves lead to diabetic complications, such as microangiopathies, nephropathies, retinopathies, peripheral neuropathies and cataract^41–43^. Consequently, aldose reductase inhibitors (ARIs) are at the focus of exploratory pharmaceutical research, as they yield the potential to prevent or control the onset of these diabetic complications. *In vitro*, AKR1B1 displays poor affinity to glucose (K_M_ = 50–100 mM) and hydrophobicity of the putative substrate-binding domain essentially precludes efficient carbohydrate reduction^44^. Therefore, lipid peroxidation-derived hydrophobic aldehydes, such as 4-hydroxynonenal (HNE) and other related nephro- and hepatotoxic compounds are more likely to be physiological substrates of AKR1B1. Finally, high catalytic efficiencies of AKR1B1 towards these compounds suggest an important metabolic role in the detoxification of lipid-derived aldehydes^27^^,^^44^^,^^45^. Physiologically and despite the relatively high K_M_ for glucose, however, selective AKR1B1 inhibition remains an effective treatment option for diabetic complications derived from glucose-related adducts^44^^,^^46^.

Overlapping substrate spectra are considered a major pitfall of many ARIs, as they often inhibit AKR1A1 and AKR1B1 simultaneously, thereby interfering with their central role in detoxification processes. This has raised the demand for selective inhibitors that preferentially bind to AKR1B1 and leave the activity of AKR1A1 unaffected^47–49^.

Additionally, the AKR1B subclass contains the AKR1B1 homologue AKR1B10 (71% sequence identity to AKR1B1), a NADPH-dependent oxidoreductase that converts both endo- and exogenous carbonyl group containing compounds to their corresponding alcohols^50–52^. In contrast to AKR1B1, AKR1B10 exhibits a more restricted substrate specificity towards isoprenyl aldehydes including farnesal and geranylgeranial, which are reduced to their corresponding alcohol metabolites^52^.

These metabolites are involved in protein prenylation, a process suspected of being crucial for carcinogenesis^28^. Moreover, AKR1B10 participates in the reduction of retinal to retinol, and thereby balances the homeostasis of retinoic acid, which is able to modulate cell proliferation and differentiation^53^^,^^54^, as well as the reductive metabolism of carbonyl group containing exogenous compounds^50–52^.

AKR1B10 is physiologically expressed in the small and large intestine and in the adrenal glands^55^^,^^56^. AKR1B10 overexpression has been reported in several types of cancer, including lung^57^^,^^58^, pancreatic^26^^,^^28^^,^^59^ and hepatocellular tumors^24^. At the same time, AKR1B10 fulfills a regulatory role in cell proliferation and differentiation by modulating the metabolism of retinoids and prenylation of oncoproteins, including Kras^26^^,^^59^. Since AKR1B10 overexpression is already detectable in precancerous lesions^50^^,^^60^, metabolic events mediated by this enzyme could play a crucial role in the development of cancer. In particular, membrane proteins like Ras and Ras-related GTP-binding proteins rely on prenylation to exert their functions in processes of cellular growth and differentiation^28^^,^^61^^,^^62^. Within the metabolism of farnesal and geranylgeranial whose reduced metabolites are important intermediates of cholesterol synthesis and protein prenylation, AKR1B10 performs key reactions by reducing farnesyl and geranylgeranial to farnesol and geranylgeraniol. The latter are further phosphorylated to farnesyl and geranylgeranyl pyrophosphates, two main intermediates of cholesterol synthesis involved in protein prenylation^28^. Increased levels of AKR1B10 expression in neoplastic cells alterate prenylation processes and post-translational modifications of the aforementioned protooncoproteins and, hence, accelerate tumor formation^28^^,^^53^.

Thus, the finding of new potent and selective inhibitors for both AKR1B10 and AKR1B1 are current challenges in biomedical research, in order to prevent uncontrolled cell proliferation and to reduce microangiopathies, as found in diabetic complications. In this article, we investigated the inhibitory potential of three hop-derived substances on three members of the AKR superfamily (namely AKR1A1, AKR1B1 and AKR1B10). Our results provide evidence that XN, IX and 8-PN are potent inhibitors of AKR1B1 and AKR1B10, while not affecting the activity of the closely related enzyme AKR1A1.

## 2. Material and methods

### 2.1. Materials

#### 2.1.1. Chemicals and reagents

XN was a friendly gift from Dr. Klaus Kammhuber, Lfl Hop Research Center Huell (Huell, Germany). IX and 8-PN were purchased from Biomol (Hamburg, Germany). NADPH was obtained from Carl Roth GmbH+Co. (Karlsruhe, Germany). Glucose, glyceraldehyde and farnesal were purchased from Sigma-Aldrich (St. Louis, MO, USA).

### 2.2. Methods

#### 2.2.1. Preparation of recombinant proteins

The recombinant enzymes AKR1A1, AKR1B1 and AKR1B10 were prepared in an *Escherichia coli* expression system according to previously published methods: AKR1A1 was a gentle gift from Prof. Dr. Vladimir Wsol^63^; AKR1B1 was a friendly gift from Dr. Nina Kassner; information about production and purification of AKR1B10 has been published before^64^. Genetic information on the specific inserts of all obtained plasmids was verified by sequencing (MWG Eurofins). The plasmids were then transformed into *E. coli* BL21 (DE3) cells. For over-expression of 6 × His-tagged enzymes, a 400-ml culture (containing the appropriate antibiotic; plasmid dependent) was grown to an optical density of 0.6 at 600 nm at 37 °C. Protein over-expression was induced by adding isopropyl-1-thio-galactopyranoside (IPTG) to the culture medium (final concentration of 1 mM). After 3 h, cells were harvested by centrifugation (6000 g, 15 min) and re-suspended in 20 ml PBS-I buffer (20 mM NaH_2_PO_4_, 500 mM NaCl, 10 mM imidazole, pH 7.4). Cell disruption was performed by ultrasonication with cooling on ice, to avoid heating. The sample was subsequently centrifuged at 100,000 *g* at 4 °C for 1 h. The obtained supernatants, containing the target protein were purified using Ni-affinity chromatography on an ÄKTA-Purifier System (Amersham Pharmacia Biotech, Uppsala, Sweden). Purification progress was monitored by SDS-PAGE of the obtained fractions (not shown). Enzyme concentrations were determined using a Qubit 2.0 fluorometric quantitation system (Life Technologies, Carlsbad, CA, USA) according to the manufacturer’s instructions.

#### 2.2.2. Determination of inhibition parameters

Catalytic properties were determined by measuring the decrease in absorbance at 340 nm at 37 °C (Cary 100 scan photometer, Varian, Pal Alto, CA, USA). A reaction mixture without inhibitor consisted of different concentrations of substrate (see Table 1 for details), 200 μM NADPH, 0.1 M NaH_2_PO_4_ buffer (pH 7.4) and an appropriate amount of enzyme in a total assay volume of 0.8 ml. Final enzyme concentrations in the assay ranged from 583 nM (AKR1B10) to 712 nM (AKR1B1). For inhibitor selectivity studies on AKR1A1, AKR1B1 and AKR1B10 stock solutions of the inhibitors XN, XI and 8-PN were prepared in dimethyl sulfoxide (DMSO). The final concentration of DMSO in the assay was ≤0.5%. Activity measurements were started without pre-incubation by adding an appropriate amount of enzyme. When collecting data for dose–response curves, initial velocities of the glyceraldehyde reduction (concentration at K_M_; enzyme specific) in the presence of inhibitors were assayed as described above. The percentage of inhibition was calculated considering the activity in the absence of inhibitor to be 100%.

**Table 1.** IC_50_ and K_i_ values of the AKR1B1 and AKR1B10-catalysed GA reduction in the presence of the inhibitors XN, IX and 8-PN.

Enzyme	Parameter	XN	IX	8-PN
AKR1B1	IC_50_ [μM]	9.11 ± 1.02	0.57 ± 0.02	0.81 ± 0.03
	K_i_ [μM]	5.29 ± 0.95	0.17 ± 0.02	0.30 ± 0.03
AKR1B10	IC_50_ [μM]	6.56 ± 0.69	1.09 ± 0.06	0.99 ± 0.04
	K_i_ [μM]	4.56 ± 0.98	0.52 ± 0.05	0.52 ± 0.05

GA concentration is equal to the K_M_ for each enzyme: 50 μM for AKR1B1 and 4 mM for AKR1B10. Data are presented as mean ± standard deviation from at least three experiments.

XN: xanthohumol; IX: isoxanthohumol; 8-PN: 8-prenylnaringenin.

Initially, the half maximal inhibitory concentrations (IC_50_ values) were determined for each inhibitor in the presence of each enzyme, using the shared substrate glyceraldehyde (set to their specific Km; 3.6 mM, 50 µM and 4 mM for AKR1A1, AKR1B1 and AKR1B10, respectively) to assess specificity among the structurally similar members of the AKR-superfamily. For IC_50_ determination, experimental data were normalised and fitted to a sigmoidal curve as implemented in GraphPad6 (GraphPad Software Inc., La Jolla, CA, USA). Whenever tight-binding inhibition was observed, the inhibition constant K_i_ was determined by fitting inhibition data to the Morrison equation as implemented in GraphPad Prism6 (GraphPad Software Inc., La Jolla, CA, USA)^65^, using non-linear regression.

In order to verify the inhibitory potency, enzyme-specific physiological substrates for AKR1B1 (glucose, K_M_ = 32 mM) and AKR1B10 (farnesal; K_M_ = 5 µM) were used to determine inhibition parameters. Enzyme inhibition parameters were assayed as described above. The inhibition mechanism of each compound for the respective enzymes was analysed by plotting IC_50_ values at different substrate concentrations (at least five inhibitor and substrate concentrations)^65^^,^^66^. All data obtained were plotted and analysed using GraphPad Prism6 (GraphPad Software Inc., La Jolla, CA, USA).

## 3. Results

### 3.1. Determination of inhibitor selectivity

**Figure 2.**
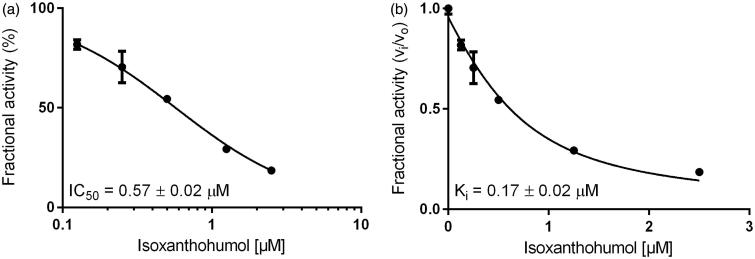
Dose–response (a) and inhibition curve of the AKR1B1-catalysed glyceraldehyde reduction by isoxanthohumol (b). Enzymatic activity is expressed as the ratio of inhibited vs. non-inhibited reaction rate. Data were fitted to the Morrison equation for tight-binding inhibitors. All data are presented as mean ± standard deviation from at least three experiments.

Initially, dose–response curves for XN, IX and 8-PN with AKR1A1, AKR1B10 and AKR1B1, using glyceraldehyde, were calculated (IC_50_- and K_i_-values are summarised in Table 1). Figure 2 exemplarily shows the determination of IC_50_- and K_i_-values for IX with AKR1B1. IX turned out to be the most effective inhibitor among the three substances for both AKR1B1 and AKR1B10 (IC_50_ = 0.57 and 1.09 µM, respectively). The IC_50_ for IX is 6 to 15 times lower than compared to XN (Table 1). Interestingly, the activity of AKR1A1 was unaffected by all three substances (IC_50_ > 50 µM).

### 3.2. Determination of inhibition parameters for physiological substrates

**Figure 3.**
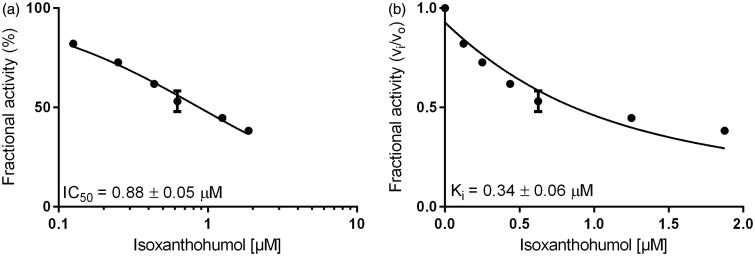
Dose–response (a) and inhibition curve of the AKR1B1-catalysed glucose reduction by isoxanthohumol (b). Enzymatic activity is expressed as the ratio of inhibited vs. non-inhibited reaction rate. Data were fitted to the Morrison equation for tight-binding inhibitors. All data are presented as mean ± standard deviation from at least three experiments.

**Figure 4.**
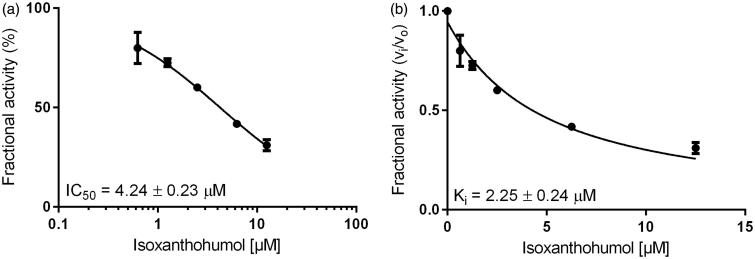
Dose–response (a) and inhibition curve of the AKR1B10-catalysed farnesal reduction by isoxanthohumol (b). Enzymatic activity is expressed as the ratio of inhibited vs. non-inhibited reaction rate. Data were fitted to the Morrison equation for tight-binding inhibitors. All data are presented as mean ± standard deviation from at least three experiments.

Inhibition constants, as well as the mode of inhibition on the reduction of two specific physiological substrates, were further calculated by using glucose for AKR1B1 and farnesal for AKR1B10, at concentrations equal to their corresponding K_M_ values (as determined before: 32 mM and 5 µM, respectively–data not shown). Figures 3 and 4 show the exemplified determination of the IC_50_- and K_i_-values (calculated using the Morrison equation^65^), in presence of IX, for AKR1B1 and AKR1B10, respectively. Tables 2 and 3 summarise the obtained IC_50_ and the inhibition constants for the respective enzymes.

**Table 2.** IC_50_ and K_i_ values of the AKR1B1-catalysed glucose reduction in the presence of the inhibitors XN, IX and 8-PN.

Enzyme	Parameter	XN	IX	8-PN
AKR1B1	IC_50_ [μM]	29.17 ± 2.30	0.88 ± 0.05	1.87 ± 0.09
	K_i_ [μM]	15.08 ± 1.65	0.34 ± 0.06	0.71 ± 0.09

Data are presented as mean ± standard deviation from at least three experiments. Glucose concentration is equal to the K_M_ (32 mM).

XN: xanthohumol; IX: isoxanthohumol; 8-PN: 8-prenylnaringenin.

**Table 3.** IC_50_ and K_i_ values of the AKR1B10-catalysed farnesal reduction in the presence of the inhibitors XN, IX and 8-PN.

Enzyme	Parameter	XN	IX	8-PN
AKR1B10	IC_50_ [μM]	41.37 ± 6.86	4.24 ± 0.23	3.96 ± 0.17
	K_i_ [μM]	20.11 ± 3.73	2.25 ± 0.24	1.95 ± 0.12

Farnesal concentration is equal to the K_M_ (5 μM). Data are presented as mean ± standard deviation from at least three experiments.

XN: xanthohumol; IX: isoxanthohumol; 8-PN: 8-prenylnaringenin.

**Figure 5.**
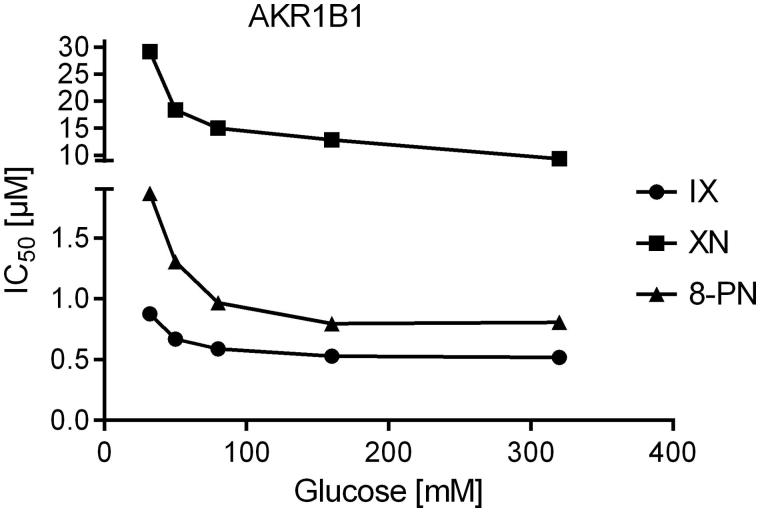
IC_50_-values of the AKR1B1-catalysed glucose reduction as a function of substrate concentration in the presence of the inhibitors xanthohumol (squares), isoxanthohumol (circles) and 8-prenylnaringenin (triangles).

**Figure 6.**
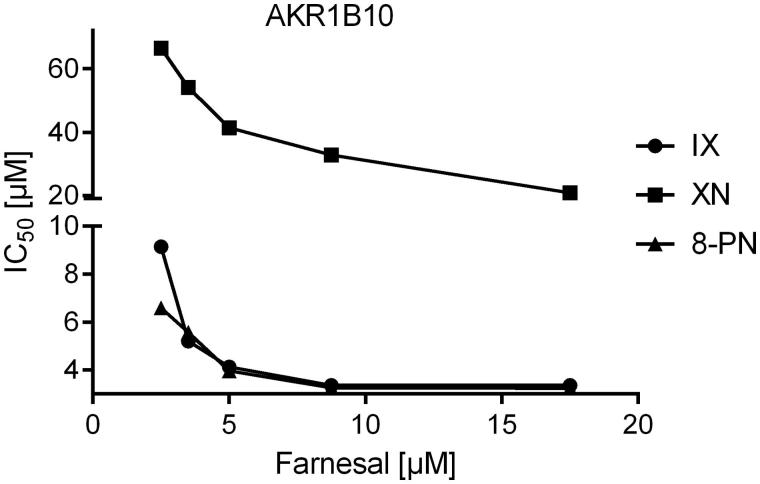
IC_50_-values of the AKR1B10-catalysed farnesal reduction as a function of substrate concentration in the presence of the inhibitors xanthohumol (squares), isoxanthohumol (circles) and 8-prenylnaringenin (triangles).

Since all three substances turned out to be tight-binding inhibitors of AKR1B1 and AKR1B10 (ratio [Inhibitor]:[Enzyme] ranging from 0.9 to 8.2), IC_50_-values were determined at five different substrate concentrations, including K_M_ to analyse the mode of inhibition. All inhibitors exhibited an uncompetitive mode of inhibition for both, AKR1B1 and AKR1B10, in the presence of their physiological substrates (Figures 5 and 6).

**Figure 7.**
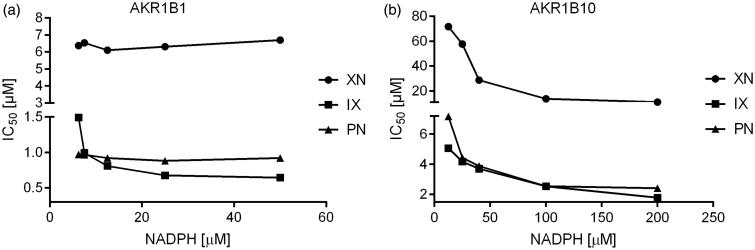
IC_50_-values of the AKR1B1-(a) and AKR1B10-(b) catalysed glyceraldehyde reduction as a function of co-substrate (NADPH) concentration in the presence of the inhibitors isoxanthohumol (squares), xanthohumol (circles) and 8-prenylnaringenin (triangles).

A closer examination of the binding mechanism of the three inhibitors was carried out by investigating their mode with respect to the co-factor binding site for NADPH. Interestingly, all the three substances behave as uncompetitive inhibitors of AKR1B10, while only IX displays the typical pattern of an uncompetitive inhibitor on AKR1B1, whereas XN and 8-PN inhibit in a non-competitive fashion (see Figure 7).

## 4. Discussion

Aldose reductase (AKR1B1) and AKR1B10 are involved in numerous pathologies and have therefore been proposed as suitable targets for drug development. Under hyperglycemic conditions, AKR1B1 promotes osmotic imbalance and modifies physiological ratios of the redox couples of both NAD^+^ and NADP^+^, with decreasing antioxidant defense abilities. Finally, it induces a potential increase in protein glycation phenomena due to an increase in AGE levels. This has led to the identification of AKR1B1 as a primary target to prevent the onset of secondary diabetic effects. AKR1B1’s homologue AKR1B10 is overexpressed in multiple cancer types, including malignancies of breast, prostate and lungs, and is deemed suitable as a target in cancer treatment. Recent studies have shown that plant/hops specific XN, IX and 8-PN act as anti-diabetic, anti-carcinogenic and antioxidative agents. Hitherto, it has not been explored how this mode of action relates to the role of AKRs in the pathogenesis of the complications described above.

Our results show that XN, IX and 8-PN are potent inhibitors of the AKR1B’s subfamily members B1 and B10 with IC_50_ and K_i_ values ranging at low µM or nM concentrations, using d,l-glyceraldehyde as a generic substrate (see Table 1). In the following, glucose (AKR1B1) and farnesal (AKR1B10) were used as corresponding physiological substrates (see Table 2). After all, IX turned out to be the most potent inhibitor on both AKR1B1 and AKR1B10, with all substrates tested (K_i_ = 0.17 μM and 0.52 μM with AKR1B1 and AKR1B10 in presence of GA as substrate and K_i_ = 0.34 μM and 2.25 μM with AKR1B1 and AKR1B10 in presence of glucose and farnesal, respectively).

Interestingly, all three inhibitors exhibit a 4′-OH group and a 2-benzyl substituent in their structure (see Figure 1). In a previous work, Rastelli et al.^67^ have shown that a 4′-OH group is crucial for the inhibition of AKR1B1 by 7-hydroxy-2–(4′-hydroxybenzil)-4H-1-benzopyran-4-one, a modified form of the potent ARI quercetin, and 2′,4,4′-trihydroxychalcone, and that it specifically binds to Thr113. The same work further showed that the 2-benzyl substituent, owing to its hydrophobic aromatic nature and conformation, optimally fits an additional hydrophobic pocket of the enzyme, lined by Trp111 and Leu300. In particular, the binding of this substituent to this additional pocket provides a selective kind of inhibition for AKR1B1 with respect to the closely related enzyme AKR1A1^67^.

There is also an evidence of prenylated flavonoids, that possess additional hydrophobic and anionic characteristic moieties (prenyl groups) at the C-8 position of their flavonoid skeletons, playing important roles in aldose reductase inhibition^68^^,^^69^. In this sense, a comparison can be made between 8-PN (AKR1B1 IC_50_ = 0.81 μM obtained in this study) and its isomer 6-prenylnaringenin (6-PN), which is also a phytoestrogenic prenylflavanone occurring in hops (AKR1B1 IC_50_ = 6.2 μM^70^). In fact, 8-PN exhibits an inhibitory potency that is 7.6 times greater as compared to that of 6-PN, applying glyceraldehyde as substrate. This might as well stress the importance of prenyl moieties at the C-8 position in terms of AKR1B1 inhibition. Although no IC_50_ data for 6-PN in presence of AKR1B10 were available at the time of this study, the low IC_50_ value obtained for 8-PN, might as well indicate a similar effect.

We further demonstrated that the three substances have no inhibitory effect on the closely related AKR member AKR1A1. This is an important result since many candidate inhibitors failed in clinical trials due to their undesired property of inhibiting both enzymes, AKR1B1 and AKR1A1^25^^,^^44^. Nevertheless, none of the inhibitors characterised in this study showed a selective inhibition, neither for AKR1B1 nor for AKR1B10. However, our studies may provide a basis for further modification of the molecules’ scaffolds, in order to design novel selective inhibitors for the respective enzymes. In fact, plenty of current studies aimed to find selective inhibitors for AKR1B1 over AKR1B10, and *vice versa*, in order to exclusively treat complications of diabetes or cancer. Yet, only a few selective inhibitors have been identified so far, including Androst-3β,5α,6β,19-tetrol (IC_50_ = 0.86 µM) or oleanolic acid (IC_50_ = 0.09 µM)^71–75^. So far, numerous tested phytogenic inhibitors have shown only moderate degrees of selectivity^71^^,^^76^.

Our results show that IX (IC_50_ = 0.57 μM for AKR1B1 and IC_50_ =  1.09 μM for AKR1B10) and 8-PN (IC_50_ = 0.81 μM for AKR1B1 and IC_50_ = 0.99 μM for AKR1B10) inhibit both enzymes with an efficacy 6 to 15 times greater than XN when applying GA as substrate (Table 1).

In presence of the physiological substrates, glucose and farnesal, the efficacy of IX (IC_50_ =  0.88 μM for AKR1B1 and IC_50_ =  0.63 μM for AKR1B10) and 8-PN (IC_50_ =  1.87 μM for AKR1B1 and IC_50_ =  3.96 μM for AKR1B10) is 10–30 times greater than that of XN (Tables 2 and 3).

IX and 8-PN are strongly related prenylated flavonoids, whereas XN is a prenylated chalcone that exhibits an open ring in its core structure, which might serve as a possible explanation for its lower inhibitory potency. Our enzyme inhibition experiments showed that all compounds under investigation exhibit an uncompetitive mode of inhibition on both, AKR1B1 and AKR1B10. Uncompetitive inhibitors exclusively bind to the enzyme-substrate complex (ES), thus circumventing competition with the substrate for the active site. For AKR1B1, this constitutes a promising mechanism of inhibition, since the affinity of uncompetitive inhibitors is greatest at saturating concentrations of the substrate, as observed under hyperglycemic conditions (>7 mM, diabetic range)^74^^,^^77^. Theoretically, under the aforementioned pathological conditions, a drastic inhibition of AKR1B1 would occur even at low inhibitor concentrations.

In order to closely examine the binding mechanism of the substances, further experiments were performed to investigate whether XN, IX and 8-PN compete with NADPH at the co-factor binding site. However, our work revealed no competition between the three tested substances and NADPH, neither for AKR1B1, nor for AKR1B10. All substances tested exhibited an uncompetitive mode of inhibition for AKR1B10 with respect to NADPH, while only IX showed an uncompetitive inhibition pattern for AKR1B1, with respect to the co-factor binding site. XN and 8-PN exhibited a non-competitive mode of inhibition on AKR1B1.

Interestingly, the abovementioned substances show an uncompetitive mode of action with respect to both, the substrate and the co-factor binding site. Hypothetically speaking, this would mean that the inhibitor does not bind to the enzyme–NADPH binary complex as expected, but, instead, to the subsequent and transient enzyme conformation after the product release but before the release of NADP+. This mechanism was previously hypothesised by Harris and Kozarich, 1997^78^ and Copeland 2005^79^ for the binding of epristeride to the NADPH-dependent steroid-5α reductase (*M. mulatta*).

Hence, XN, 8-PN, IX for AKR1B10, and IX for AKR1B1 might act as uncompetitive inhibitors that bind to an enzyme species, following the formation of the initial ES complex. More challenging to explain is the behavior of 8-PN and XN, which both have been showing a non-competitive mode of inhibition with respect to AKR1B1 at the NADPH binding site.

Since the models obtained from molecular docking experiments (data not shown) did not clarify the binding mechanism of the three compounds, further investigations by X-ray diffraction are required in future studies.

Overall, we could show that the hop compounds tested have a selective inhibitory effect on the AKR1B subfamily, with respect to AKR1A1. All substances exhibit an uncompetitive mode of inhibition on both, AKR1B1 and AKR1B10. This fact might be promising when it comes to designing novel drugs and molecular therapies with high efficacy even at low concentrations. The results of this study may also provide a basis for further quantitative structure–activity relationship models and favorable scaffolds for new selective inhibitors of AKR1B1 or AKR1B10.
